# Supplementary material for: Human Tongue Electrophysiological Response to Oleic Acid and Its Associations with PROP Taster Status and the CD36 Polymorphism (rs1761667)
Source: Nutrients. 2019 Feb 1;11(2):315. doi: 10.3390/nu11020315 (PMC6412840; doi:10.3390/nu11020315)
Supplement: Supplementary file 1 [file nutrients-11-00315-s001.pdf]

Supplemental Material:

Table S1. Ratings of perceived taste intensity in response to three concentrations of PROP and NaCl in the taster groups.

	**Super-tasters (*n* = 10)**	**Medium-tasters (*n* = 13)**	**Non-tasters (*n* = 12)**
**PROP (mmol/L)**			
0.032	10.95 ± 2.35	5.79 ± 1.72	0.66 ± 0.33
0.32	53.33 ± 6.20 *	35.23 ± 3.75	3.95 ± 1.02 *
3.2	91.50 ± 3.07 *	57.31 ± 3.92	28.84 ± 4.93 *
**NaCl (mol/L)**			
0.01	2.18 ± 0.72	8.63 ± 2.67	3.52 ± 1.42
0.1	26.55 ± 5.66 *	33.01 ± 5.05	28.50 ± 4.52 *
1	58.67 ± 5.95 *	64.49 ± 6.63	58.50 ± 4.75 *

Values are means ± SEM. $n = 35$. Three-way ANOVA was used to compare PROP intensity ratings with NaCl intensity ratings across groups ($F_{(4,192)} = 5.302$; $p = 0.00045$).
* Significant difference between PROP and the corresponding NaCl concentration ($p < 0.00022$; Newman Keuls test).

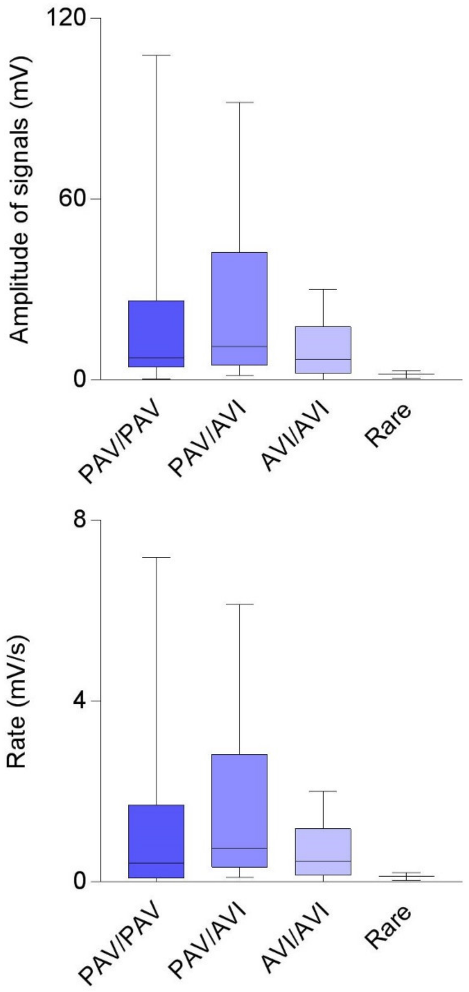

Figure S1. Box-and-whisker plots showing the minimum, first quartile, median, third quartile, and maximum of each data set of amplitude and rate of signals evoked by oleic acid (30 μl) taste stimulation in individuals with genotypes PAV/PAV (n = 7), PAV/AVI (n = 16), AVI/AVI (n = 10) and rare genotypes (n = 2) of *TAS2R38*. One-way ANOVA showed no difference of amplitude and hyperpolarization rate related to the *TAS2R38* genotype ($p > 0.05$).

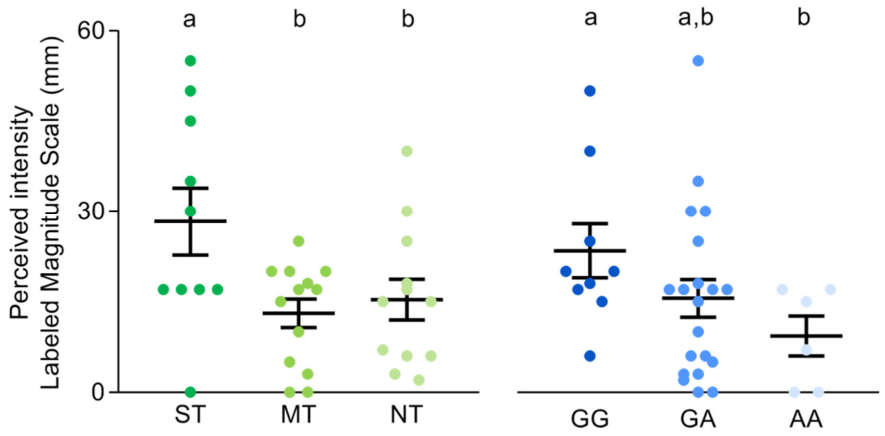

Figure S2. Distribution of data of the perceived intensity after taste stimulation with oleic acid (30 µl) in super-tasters (ST; n = 10), medium-tasters (MT; n=13) and non-tasters (NT; n = 12), and in volunteers with genotypes GG (n = 9), GA (n = 20) and AA (n = 6) of *CD36*. Mean values ± SEM are also shown. Different letters indicate a significant difference (p ≤ 0.046; Duncan's test and p = 0.047 Fisher LDS test, subsequent one-way ANOVA).

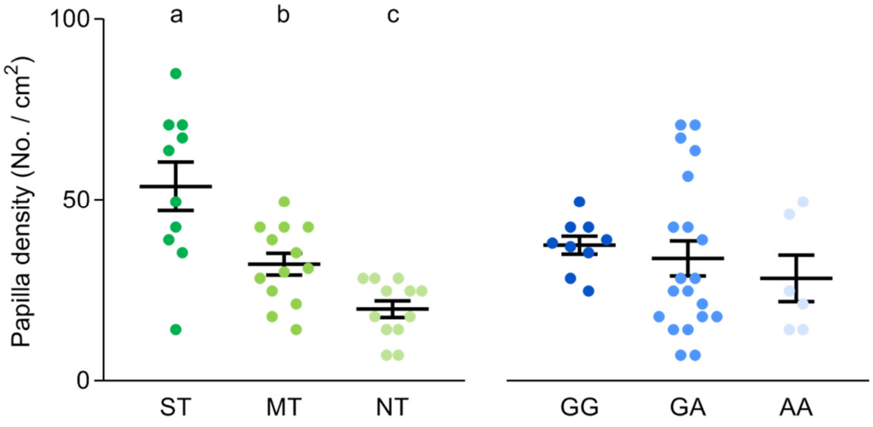

Figure S3. Distribution of data of the density of fungiform papillae in super-tasters (ST; n = 10), medium-tasters (MT; n=13) and non-tasters (NT; n = 12), and in volunteers with genotypes GG (n = 9), GA (n = 20) and AA (n = 6) of *CD36*. Mean values ± SEM are also shown. Different letters indicate a significant difference (p ≤ 0.032; Duncan's test subsequent one-way ANOVA).

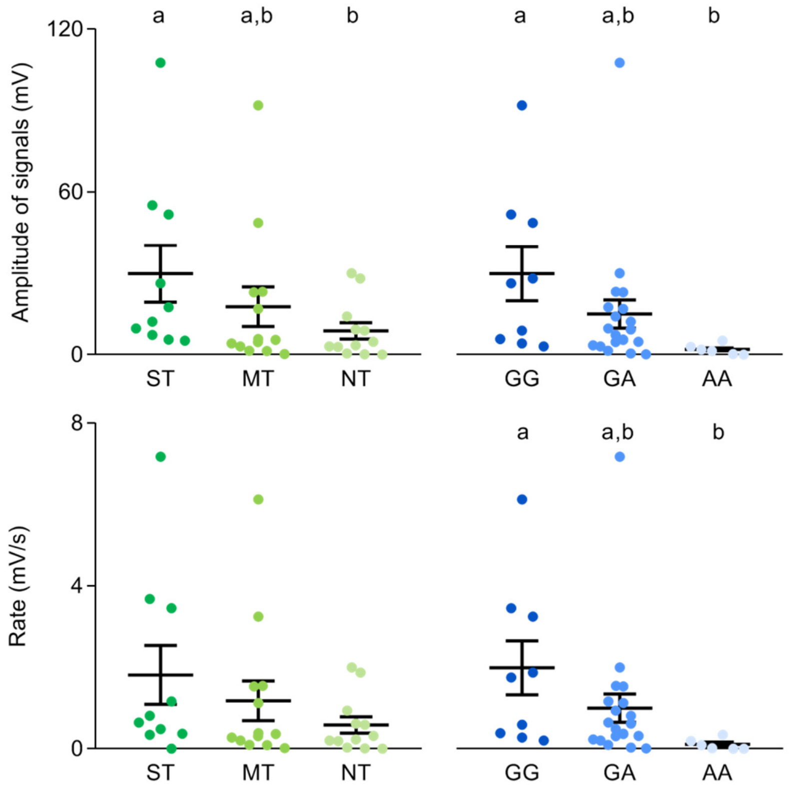

Figure S4. Distribution of data of amplitude and rate of signals evoked in super-tasters (ST; n = 10), medium-tasters (MT; n=13) and non-tasters (NT; n = 12), and in volunteers with genotypes GG (n = 9), GA (n = 20) and AA (n = 6) of *CD36*. Mean values ± SEM are also shown. Different letters indicate a significant difference ($p \leq 0.05$; Fisher LDS or Duncan's test subsequent one-way ANOVA).
